# Baseline identification of clonal V(D)J sequences for DNA-based minimal residual disease detection in multiple myeloma

**DOI:** 10.1371/journal.pone.0211600

**Published:** 2019-03-22

**Authors:** Even H. Rustad, Malin Hultcrantz, Venkata D. Yellapantula, Theresia Akhlaghi, Caleb Ho, Maria E. Arcila, Mikhail Roshal, Akshar Patel, Denise Chen, Sean M. Devlin, Austin Jacobsen, Ying Huang, Jeffrey E. Miller, Elli Papaemmanuil, Ola Landgren

**Affiliations:** 1 Myeloma Service, Department of Medicine, Memorial Sloan Kettering Cancer Center, New York, NY, United States of America; 2 Department of Clinical and Molecular Medicine, Faculty of Medicine and Health Sciences, Norwegian University of Science and Technology, NTNU, Trondheim, Norway; 3 Department of Epidemiology and Biostatistics, Memorial Sloan Kettering Cancer Center, New York, NY, United States of America; 4 Department of Pathology, Memorial Sloan Kettering Cancer Center, New York, NY, United States of America; 5 Center for Hematological Malignancies, Department of Medicine, Memorial Sloan Kettering Cancer Center, New York, NY, United States of America; 6 Marie-Josee and Henry R. Kravis Center for Molecular Oncology, Memorial Sloan Kettering Cancer Center, New York, NY, United States of America; 7 Invivoscribe, Inc, San Diego, CA, United States of America; Leiden University Medical Centre, NETHERLANDS

## Abstract

Tracking of clonal immunoglobulin V(D)J rearrangement sequences by next generation sequencing is highly sensitive for minimal residual disease in multiple myeloma. However, previous studies have found variable rates of V(D)J sequence identification at baseline, which could limit tracking. Here, we aimed to define the factors influencing the identification of clonal V(D)J sequences. Bone marrow mononuclear cells from 177 myeloma patients underwent V(D)J sequencing by the LymphoTrack assays (Invivoscribe). As a molecular control for tumor cell content, we sequenced the samples using our in-house myeloma panel myTYPE. V(D)J sequence clonality was identified in 81% of samples overall, as compared with 95% in samples where tumor-derived DNA was detectable by myTYPE. Clonality was detected more frequently in patients with lambda-restricted disease, mainly because of increased detection of kappa gene rearrangements. Finally, we describe how the tumor cell content of bone marrow aspirates decrease gradually in sequential pulls because of hemodilution: From the initial pull used for aspirate smear, to the final pull that is commonly used for research. In conclusion, baseline clonality detection rates of 95% or higher are feasible in multiple myeloma. Optimal performance depends on the use of good quality aspirates and/or subsequent tumor cell enrichment.

## Introduction

Achieving minimal residual disease (MRD) negativity after initial treatment for multiple myeloma is strongly associated with prolonged progression free (PFS) and overall survival (OS)[1–5]. Consequently, MRD testing is done in the vast majority of clinical trials, and it is gradually becoming standard of care. The clinical implications of MRD negativity are rapidly evolving as more sensitive methods are introduced. When MRD was first included in the IMWG response criteria for multiple myeloma in 2016, MRD negativity was defined as less than 1 tumor cell in 100 000 bone marrow cells (i.e. 10^−5^)[2]. Emerging evidence now shows prolonged PFS when raising the bar for MRD negativity to 10^−6^ (i.e. less than 1 tumor cell in 1 000 000 bone marrow cells), compared to the current IMWG definition [6–8].

Two main methods of MRD assessment are used today: Flow cytometry and DNA-based next generation sequencing (NGS). Flow cytometry has been the standard method because of its wide availability in hematology laboratories and applicability in almost all patients [9, 10]. According to the literature, the two most sensitive flow cytometry assays are: (1) the EuroFlow 8-color 2-tube panel, with a reported analytical sensitivity of 2 tumor cells in 1 000 000 (10^−6^) bone marrow cells if the recommended 10 million cells can be acquired; and (2) the Memorial Sloan Kettering Cancer Center 10-color single tube panel, requiring 3 million cell acquisitions to reach a sensitivity of 6 tumor cells in 1 000 000 [11, 12]. However, there is lack of standardization across laboratories, concerning antibody panels, gating strategies and the number of cells analyzed [13, 14]. This translates into variable sensitivity of MRD testing, which is most often between 1 tumor cell in 10 000 (10^−4^) bone marrow cells, and 1 in 100 000 cells (10^−5^) [3, 13, 14].

NGS-based MRD assays have an analytical sensitivity of 10^−6^, contingent on DNA input from 3 million bone marrow cells [6, 15, 16]. In contrast to flow cytometry, NGS-assays do not require fresh samples; and the workflow can be fully automated from sample preparation to analysis, thus avoiding inter-observer variation [9, 17]. NGS-based assays are based on capturing the dominant clonal immunoglobulin heavy chain (*IGH*) or kappa light chain (*IGK*) V(D)J rearrangement at baseline, which is then tracked by ultra-deep sequencing in post-treatment samples [9, 15]. Rates of baseline V(D)J sequence identification vary from 80 to 97% in the literature, with most studies around 90–93% [5–8, 11, 17–21]. Optimizing the detection of V(D)J sequence clonality at baseline is important to ensure patients access to highly sensitive MRD assessment; however, the factors underlying failure to identify the baseline clone are poorly understood.

We were motivated to conduct a study designed to address V(D)J sequence clonality in a large cohort of plasma cell myeloma samples. In particular, we aimed to define the sample-related and disease-related factors underlying the success or failure to identify the clonal V(D)J sequence. To accomplish this, we compared the results of V(D)J sequencing to a multiple myeloma NGS panel as a molecular control for the tumor cell content of each sample. This allowed us to dissect out the effects of biological factors on clonality detection, including immunoglobulin class and the potential role of somatic hypermutation (SHM) of the immunoglobulin variable region.

## Methods

### Patients and clinical data

We included baseline bone marrow aspirates from 177 patients with plasma cell myeloma. Patient characteristics at the time of sampling were obtained by chart review. When available we also extracted data on immunophenotype by flow cytometry. Retrospective chart review and use of stored biospecimens for research was approved by the Memorial Sloan Kettering Cancer Center Institutional Review Board (09–141, 06–107, 14-276A(6) and 15–017) and all patients provided written informed consent.

### Sample preparation

Bone marrow mononuclear cells (MNCs) were isolated by ficoll density gradient separation. In 7 cases, MNCs were further processed by magnetic bead separation of CD138+ plasma cells, using a MACSR Separator.

### Immunoglobulin heavy chain and kappa light chain gene sequencing with LymphoTrack

Identification of clonal V(D)J sequences by the LymphoTrack assays (Invivoscribe Inc, San Diego, CA) follows a three-step workflow: 1) PCR amplification, 2) NGS and 3) bioinformatics analysis. For the PCR step, we used five LymphoTrack assays: *IGH* FR1, *IGH* FR2, *IGH* FR3 (FR 1–3 assay catalogue number #7-121-0139), *IGK (*#7-122-0019) and *IGH* Leader (#7-121-0069). Each assay has a single multiplex master mix that targets conserved regions in the *IGH* or *IGK* genes. In brief, each of the IGH master mixes consists of forward primers targeting variable (V_H_) region framework regions 1 to 3 (FR1, 2, 3) as well as several consensus reverse primers targeting the joining (J_H_) region. The fourth IGH assay “Leader” is designed for use in cases where SHM prevents primer annealing to FR regions, with a master mix consisting of forward primers targeting the leader sequences upstream of the V_H_ region. The downstream primers are the same consensus (J_H_) primers used in the other IGH assays. Finally, the *IGK* assay contains forward primers targeting conserved variable (V_K_) region and intron sequences, with reverse primers targeting joining (J_K_) and kappa deleted element (Kde) regions. NGS was done a MiSeq instrument (Illumina, San Diego, CA) using the MiSeq Reagent Kit v3 (600-cycle) with 2×251 bp length for all assays, except Leader, which required 2×301 bp length. Bioinformatics analysis to detect and quantify of unique rearrangement sequences was done using commercially available LymphoTrack software (Invivoscribe). The result of each assay was called as clonal, non-clonal or indeterminate (i.e. too few reads for evaluation) according to standardized criteria developed from validation experiments (Supplemental Methods in S1 Appendix). Detailed step-by-step protocols for wet-lab as well as bioinformatics analysis are available on the Invivoscribe website.

In this study, *IGH* FR1-3 and *IGK* was run first on all samples, and the Leader assay was reflexed if the results of FR1 was indeterminate and no clonal V(D)J sequence was identified by another assay. Input material was 50 ng of DNA for each assay, with a target coverage of > 20 000 X.

SHM of clonal V_K_ rearrangements was quantified using the web-based IgBLAST tool with ImMunoGeneTics (IMGT) germline reference databases [22]. Significant SHM was defined as >2% variation from the germline sequence, according to standard practices.

### Somatic variant detection with myTYPE

The myTYPE NGS assay was designed to capture all recurrent genomic aberrations in multiple myeloma. This assay uses solution phase hybridization-based exon capture and massively parallel DNA sequencing to capture all protein-coding exons and select introns of 120 genes previously described for recurrent single nucleotide variants (SNVs) and small insertions/deletions (indels) in multiple myeloma and other hematological malignancies (oncogenes, tumor suppressor genes, and members of pathways deemed actionable by targeted therapies). In addition, we captured the entire IGH locus where the vast majority of the chromosome 14 translocation breakpoints occur, as well as genome wide single nucleotide polymorphisms (1 per 3 Mb) to assess hyperdiploidy and other copy number aberrations (CNAs).

Barcoded sequence libraries (New England Biolabs, Kapa Biosystems, Wilmington, MA, USA) were subjected to exon capture by hybridization (Nimblegen SeqCap, Madison, WI, USA). 100 to 200 ng of gDNA was used as input for library construction. Libraries were pooled at equimolar concentrations (100 ng per library) and input to a single exon-capture reaction as previously described[23]. To prevent off-target hybridization, a pool of blocker oligonucleotides complementary to the full sequences of all barcoded adaptors was spiked in to a final total concentration of 10 μmol/L. DNA was subsequently sequenced on an Illumina HiSeq 4000 to generate paired-end 100-bp reads. Sequence data were demultiplexed using CASAVA, and reads were aligned to the reference human genome (hg19) using the Burrows-Wheeler Alignment tool (12). Samples were sequenced to a median coverage of 600x for tumor samples and 250x for normal samples, with a minimum of 99% of the targeted region covered 30 fold.

Putative somatic variants were identified using in-house algorithms followed by manual review (described in detail in Supplemental Methods in S1 Appendix). Briefly, substitutions and small insertions/deletions were manually annotated as oncogenic, likely oncogenic, unknown, artifact or germline SNP based on genetic criteria, as previously described [24]. CNAs were identified based on manual review of copy number plots. Translocations were called as real events if they were supported by high quality reads and involved the IGH locus on chromosome 14 as well as a partner commonly described in multiple myeloma. Validation of myTYPE in samples enriched for CD138+ tumor cells showed good concordance with whole genome sequencing, and we detected more translocations and CNAs compared with standard fluorescent in situ hybridization [25]. In this study, samples were considered as myTYPE positive if the above analyses identified at least one high confidence somatic variant known to be recurrent in myeloma.

Final manually curated SNVs and insertions/deletions have been deposited to the European Variation Archive (EVA) with accession numbers PRJEB31370 (project) and ERZ807140 (analyses). Structural variants and copy number changes are included in S1 File.

### Statistics

To define predictors of V(D)J sequence identification, we conducted univariate logistic regression analysis of all candidate predictors. Results are presented as odds ratios (ORs) with 95% confidence intervals (CIs). Statistically significant predictors from the univariate analysis were selected for a multivariate model. Chi-squared test was used for contingency tables, and Mann-Whitney U test for group comparisons, unless otherwise specified. Two-sided p-values below 0.05 were considered statistically significant. Data analysis was done in R version 3.4.3.

## Results

### LymphoTrack assay validation

To assess the performance of the LymphoTrack *IGH* FR1-3, *IGH* Leader and *IGK* assays, we performed serial dilution experiments of clonal control DNA in polyclonal tonsil DNA, followed by statistical determination of the assay limit of detection. All assays could distinguish clonal sequences from the background down to 2.5% of reads, contingent on 20 000 X or higher target coverage (S1 Fig and S1 Table). Algorithms to determine sample clonality at various sequencing depths were developed accordingly (S2, S3 and S4 Figs). Finally, the validity of the assay and clonality criteria was confirmed by comparison with capillary electrophoresis in 59 heterogeneous de-identified clinical samples, showing > 95% concordance (S2 Table).

### Clonality in patients with plasma cell myeloma

Baseline bone marrow aspirates from 177 patients with plasma cell myeloma were sequenced with LymphoTrack to identify the clonal V(D)J rearrangement sequence. At the time of baseline sampling, 26 patients had smoldering multiple myeloma (SMM), 104 had newly diagnosed multiple myeloma (NDMM), and 47 had relapsing or refractory multiple myeloma (RRMM) according to IMWG criteria[2, 26]. Among patients with SMM, the median bone marrow plasma cell infiltration by bone marrow aspirate was 12% (quartile range 8–20)(Table 1). For NDMM and RRMM, the corresponding numbers were 24% (12–44) and 25% (13–40), respectively. Demographics and immunoglobulin classes were similar in SMM, NDMM and RRMM (Table 1). Clonality was detected in 81% of samples overall (Table 2), which motivated us to look further into the underlying reasons for failure to identify the clonal sequence.

**Table 1:** Patient characteristics at the time of V(D)J sequencing.

	Smoldering multiple myeloma	Newly diagnosed multiple myeloma	Relapsing/ refractory multiple myeloma
**Number of patients**	26	104	**47**
Gender (male)*	12 (46%)	55 (53%)	**25 (53%)**
Age at sampling (years)**	63 (55.25–75)	63 (55.75–71)	**62 (57.5–67.5)**
PC % on BM aspirate differential**	12% (8–19.75)	23.5% (11.75–44)	**25% (12.5–40)**
PC % on BM biopsy**	13% (10–20)	50% (20–80)	**37.5% (15–67.5)**
Monoclonal PC as % of WBC by flow of BM**	0.55% (0.2–2.575)	2% (0.3–11)	**2.4% (0.4–5.6)**
Ig heavy chain*			
**IgG**	20 (77%)	61 (59%)	**28 (60%)**
**IgA**	4 (15%)	28 (27%)	**8 (17%)**
**IgD**	0 (0%)	2 (2%)	**0 (0%)**
**None**	2 (8%)	13 (12%)	**11 (23%)**
Ig light chain*			
**Kappa**	15 (58%)	71 (68%)	**27 (57%)**
**Lambda**	11 (42%)	33 (32%)	**20 (43%)**
**None**	0 (0%)	0 (0%)	**1 (2%)**
Serum M-spike (g/dl)**	0.9 (0.4–1.6)	2.2 (0.8–3.2)	**1.1 (0.2–2)**
Involved s-FLC (mg/dl)**	11.2 (2.8–16)	38 (9.6–115.6)	**26.8 (3.4–94)**
Ratio of involved to uninvolved s-FLC**	**13 (5–30)**	**59 (12–214.5)**	**80 (9–686)**

Patients were grouped by disease stage according to IMWG criteria[2, 26]. Variables marked by * are reported as number of patients (%)

** as median (quartile range). PC, plasma cells; BM, bone marrow; WBC, white blood cells; Ig, immunoglobulin; s-FLC, free light chains.

**Table 2:** Clonality detection overall and in myTYPE positive patients.

	All (n = 177)	Smoldering multiple myeloma (n = 26)	Newly diagnosed multiple myeloma (n = 104)	Relapsing/ refractory multiple myeloma (n = 47)
Clonality overall	144 (81%)	19 (73%)	84 (81%)	41 (87%)
myTYPE positive	76/169 (45%)	7/24 (29%)	42/98 (43%)	27/47 (57%)
Clonality in myTYPE pos	72 (95%)	6 (86%)	42 (100%)	24 (89%)

V(D)J sequencing was done on all samples; myTYPE was done on all samples with sufficient remaining DNA. Numbers and percentages of positive calls are shown for the whole cohort as well as subgroups.

### High rates of clonality where the presence of tumor-derived DNA was confirmed by an independent assay

To determine the effect of tumor cell content in the patient samples on clonality detection, we estimated the tumor cell content by two parallel approaches: 1) indirectly, based on the percentage of plasma cells by morphologic assessment of the corresponding aspirate smear, and 2) directly, by measuring the presence of tumor-derived DNA in the sample with another NGS panel as a ‘molecular control’. For this purpose we used the targeted sequencing assay myTYPE, which was designed to detect all the recurrent genomic aberrations in multiple myeloma.

**Fig 1:**
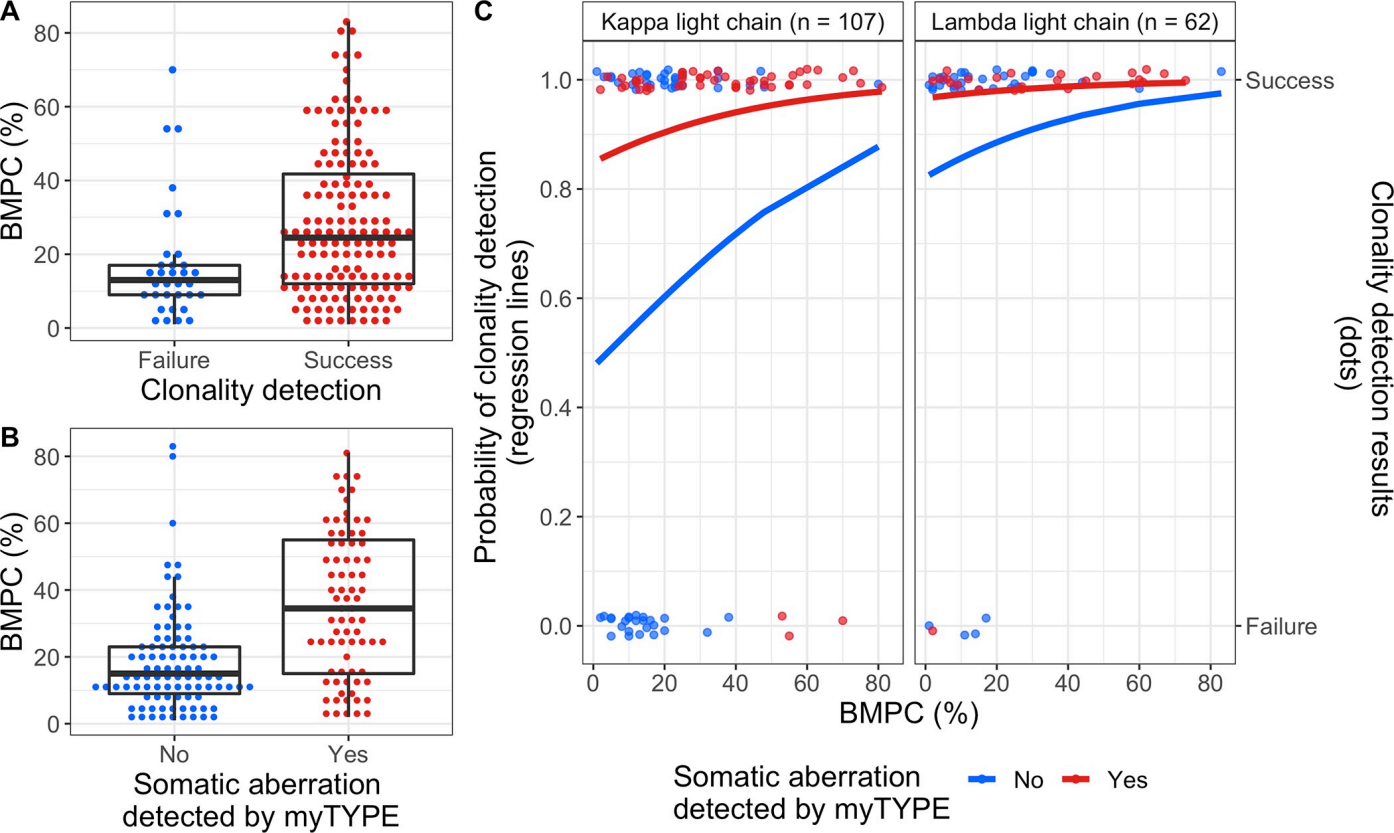
Predicting clonality detection. **A:** Dot plot showing the percentage of bone marrow plasma cells in samples by success or failure of clonality detection. Median and quartile range are shown as a superimposed boxplot. **B**: Similar plot as in A, showing bone marrow plasma cells in samples where myTYPE was positive versus negative. **C:** Individual sample data and regression curves from a multivariate model to predict clonality detection. The right panel shows lambda light chain restricted multiple myeloma; the left panel shows kappa light chain multiple myeloma, including 1 patient whose tumor cells are negative for both kappa and lambda staining. Within each panel, regression lines shows the probability of clonality detection as a function of bone marrow plasma cell infiltration by aspirate smear within the myTYPE positive (red) and negative (blue) groups. Points plotted along the top panel border represent samples where clonality was detected, whereas no clonal V(D)J sequence could be found in the samples at the bottom. BMPC, plasma cells in bone marrow aspirate smear.

Bone marrow plasma cell infiltration was higher in samples where clonality was detected, but there was also extensive overlap between the groups: (24% (quartile range 12–41.7) versus 13% (9–17); p = 0.002) (Fig 1A). Sufficient DNA for myTYPE analysis was available for 169 out of 177 samples. While we would expect ~100% of patients to be myTYPE positive (i.e. have at least one detectable genomic aberration)[25, 27, 28], this was only the case for 45% of samples in our cohort (Table 2). In the remaining samples, the results of myTYPE were either normal, or putative somatic variants fell below the threshold where we could confidently separate real variants from sequencing noise. Positive myTYPE was associated with higher plasma cell infiltration in the corresponding aspirate smear, but with significant between-group overlap, similar to what we found for clonality detection (43% (15–55) versus 15% (9–23); p < 0.001)(Fig 1B). Interestingly, in samples with confirmed presence of tumor-derived DNA by myTYPE, the success rate of clonality detection was 95%, as compared with 70% when myTYPE was negative (McNemar’s test, p < 0.001)(Table 2). This reveals low tumor cell content as the main reason for failure to identify a clonal V(D)J sequence in our study.

No clonal V(D)J sequence could be identified in four samples (5%) despite positive myTYPE, demonstrating the presence of biological factors interfering with clonality analysis in a minority of cases. All of these patients had bone marrow infiltration by kappa-restricted plasma cells; one had smoldering multiple myeloma and the remaining three had relapsed/refractory disease.

### Tumor cell content and light chain restriction are independent predictors of clonality detection

Next, we set out to determine which factors have independent effects on clonality detection, using logistic regression analysis. The strongest individual predictors from univariate regression were combined in a multivariate model (Table 3 and S3 Table). We also evaluated the immunophenotype of plasma cells by flow cytometry, which did not differ significantly by success versus failure of clonality analysis (S4 Table).

**Table 3:** Prediction models for clonality detection.

	Univariate models			Multivariate model		
Predictor	n	Odds ratio (95% CI)	p-value	n	Odds ratio (95% CI)	p-value
Bone marrow plasma cells by aspirate smear (per 10% increase)	177	1.44 (1.13–1.91)	0.006	167	1.3 (0.97–1.83)	0.110
myTYPE positive	169	7.75 (2.86–27.24)	<0.001	167	6.20 (2.08–23.3)	0.002
Lambda light chain restriction	177	4.00 (1.58–12.31)	0.007	167	5.10 (1.89–16.49)	0.003

Here, we show the three main predictors for V(D)J capture in univariate and multivariate logistic regression models. In the multivariate model, the odds ratio and p-value represents the effect of the predictor on V(D)J capture while the other predictors were held constant. Bone marrow plasma cell infiltration was analyzed as continuous variable, with odd ratio and 95% CI reported for 10% increments.

Multivariate regression confirmed myTYPE positivity as a strong independent predictor clonality detection (OR 6.20, 95% CI 2.08–23.3, p = 0.002) (Table 3, Fig 1). Plasma cell content estimated from a bone marrow aspirate smear also contributed to the multivariate model, with an OR of 1.3 for each 10% increase in plasma cell content (95% CI 0.97–1.83, p = 0.110), but this did not reach statistical significance after accounting for the strong effect of myTYPE. Finally, having lambda light chain restricted plasma cells was a strong independent predictor for clonality detection (OR 5.10, 95% CI 1.89–16.49, p = 0.003). This effect was independent of the heavy chain class.

### Frequent identification of clonal *IGK* rearrangements in lambda-restricted multiple myeloma

**Fig 2:**
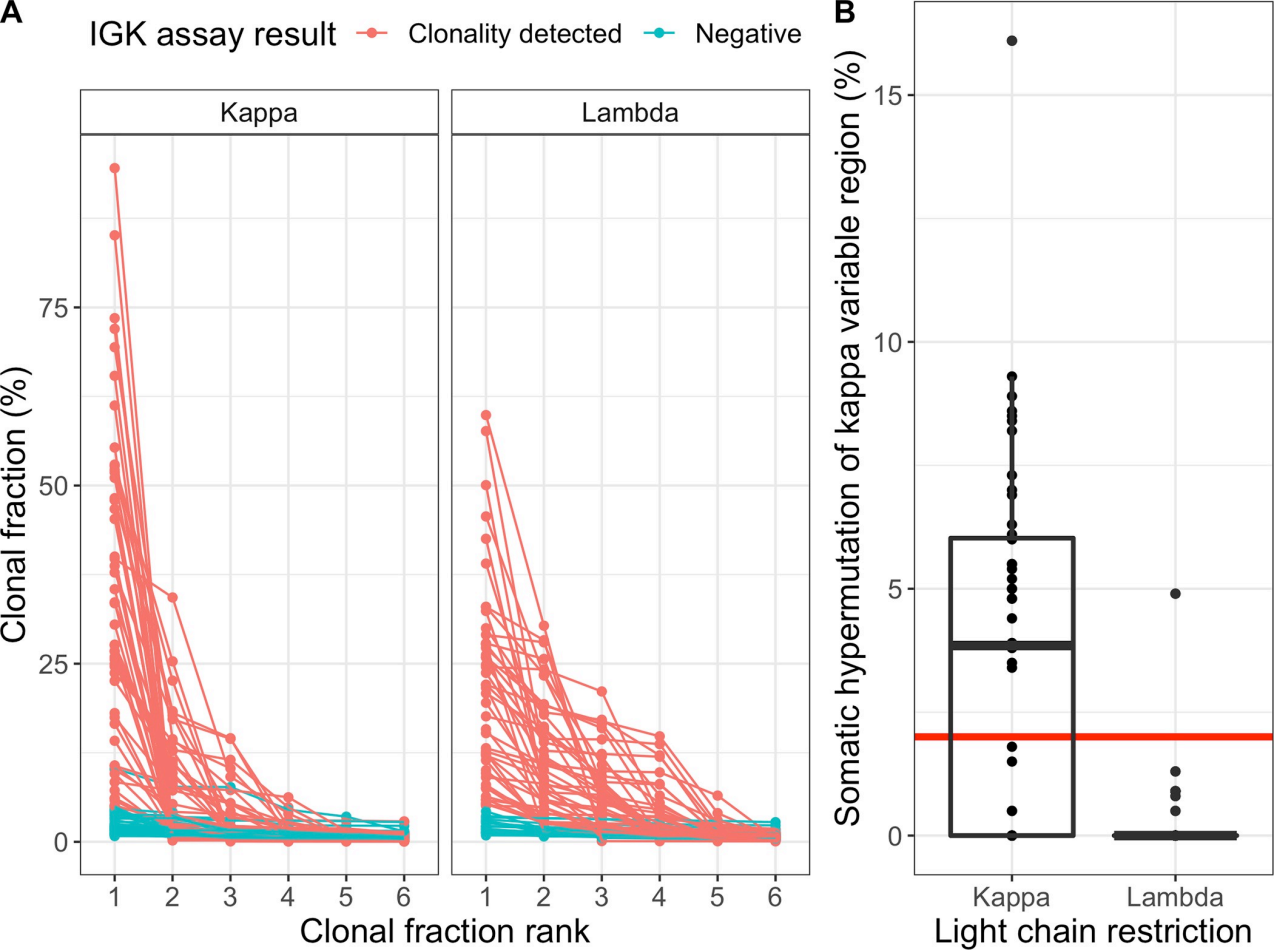
More *IGK* rearrangements and minimal somatic hypermutation of clonal V_K_-sequences in lambda-restricted multiple myeloma. **A:** Clonal fractions of the six most abundant *IGK* rearrangements in kappa-restricted cases are shown to the left; lambda-restricted to the right. Lines connect rearrangements derived from the same sample. Samples are colored red where the most abundant rearrangement was defined as clonal by the analysis software. Samples where no clonal rearrangement could be identified (teal) are shown to give an impression of the polyclonal background. Lambda-restricted cases show evidence of more clonal sequences compared with kappa-restricted. **B:** SHM of the most abundant clonal rearrangements involving the V_K_ region in kappa-restricted and lambda-restricted cases, measured as % change from the germline sequence. The red horizontal line at 2% represents the commonly used cut-off between sequences considered non-mutated (below) as compared with significantly mutated (above).

Prompted by the higher rate of clonality in lambda-restricted multiple myeloma, we searched for an explanation in the performance of individual assays targeting *IGH* variable region frameworks 1, 2 and 3; as well as the *IGK* assay (Table 4). This revealed a significantly higher success rate of the *IGK* assay in patients with lambda-restricted multiple myeloma as compared with kappa-restricted (72 vs. 45%, p = 0.0007). To elucidate the underlying mechanisms for this observed difference, we considered the specific *IGK* rearrangements identified, as well as the degree of SHM in clonal V_K_ sequences. V_K_J_K_ rearrangements were the most common major clones identified in both lambda- and kappa-restricted multiple myeloma (60 vs. 78%), whereas inactivating rearrangements involving the kappa-deleted element (Kde) were more common with lambda-restriction (34 vs. 16%)(S5 Table). In samples where a clonal *IGK* rearrangement had been identified, additional rearrangements of likely tumor origin were detected more frequently in lambda-restricted cases (up to four per sample) compared with kappa-restricted (Fig 2A). This is consistent with the current paradigm of *IGK* inactivation, where virtually all inactivated alleles contain a rearrangement of Kde, with or without an additional V_K_J_K_ rearrangement [29–31]. All of these rearrangements are potentially amenable to detection by the LymphoTrack *IGK* assay. Finally, significant SHM was present in only 3%, (1/33) of clonal V_K_- rearrangements in lambda-restricted cases, compared with 58% (28/48) of kappa-restricted (p < 0.001) (Fig 2B). Taken together, the presence of several unique rearrangements and minimal SHM provides favorable conditions for identifying clonal *IGK* rearrangement sequences in lambda-restricted plasma cells.

**Table 4:** Success rate of clonality assays overall and in subgroups.

Assay usage	All (n = 177)	myTYPE positive (n = 76)	Lambda (n = 64)	Kappa (n = 113)
All	144 (81%)	72 (95%)	60 (92%)	84 (75%)
*IGH* FR1	84 (47%)	46 (61%)	32 (49%)	52 (46%)
*IGH* FR2	80 (45%)	49 (64%)	31 (48%)	49 (44%)
*IGH* FR3	85 (48%)	43 (57%)	35 (54%)	50 (45%)
*IGK*	97 (55%)	58 (76%)	47 (72%)	50 (45%)

Capture assays for *IGH* variable region frameworks (FR) 1, 2 and 3 as well as *IGK* were performed on all samples. Number and percentage of successful captures is shown for each assay in all samples (first column) or various subgroups. The overall capture rate in lambda-restricted cases was significantly higher than in kappa-restricted cases. This was also the case for the *IGK* assay alone, but not for each of the *IGH* assays. The *IGH* Leader assay was performed on 13 samples, of which 5 were positive. These results are included in capture rate for all assays combined.

### Gradient of plasma cell content in bone marrow samples

**Fig 3:**
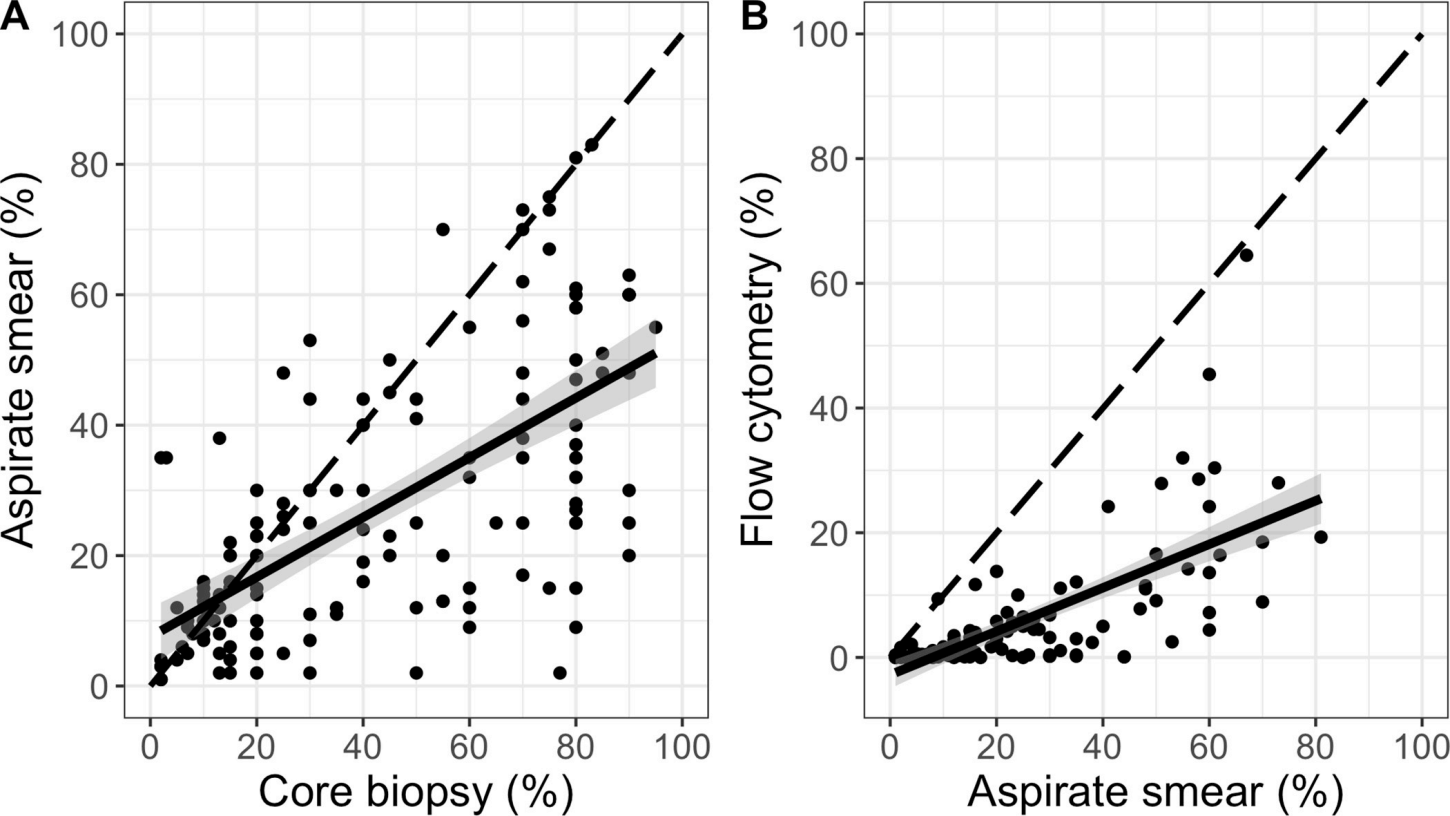
Progressively lower estimates of plasma cell content from core biopsy through aspirate smear and flow cytometry. **A:** Dot plot of bone marrow plasma cell infiltration by aspirate smear versus core biopsy, with a fitted linear regression line (solid black) surrounded by its 95% confidence interval (n = 146; slope 0.46; 95% CI 0.37–0.55; R^2^ = 0.428; p<0.001). **B:** Similar plot as in A, showing aspirate smear and flow cytometry (n = 114; slope 0.35; 95% CI 0.28–0.42; R^2^ = 0.483; p<0.001). The diagonal dashed line shows where dots would have aligned if there was a 1:1 relationship between measurements.

Because the plasma cell content in samples is essential to detect clonality, we compared the three ways by which this is measured in clinical practice: Core biopsy, aspirate smear, and flow cytometry. The plasma cell infiltration in a bone marrow aspirate smear was on average 46% that of the core biopsy (Fig 3A), while flow cytometry provided a progressively lower estimate, at 35% of the aspirate smear (Fig 3B). This plasma cell concentration gradient is well known from clinical practice and can be explained by differing degrees of peripheral blood contamination (hemodilution) of bone marrow aspirates, as well as other factors [32]. Core biopsies directly represent the plasma cell content of the bone marrow and are considered gold standard. Morphologic assessment of bone marrow aspirate smear is the second most accurate, as it is commonly obtained from the first aspirate pull. The lower plasma cell content in aspirate smears compared with core biopsies can be explained by hemodilution of the aspirate, as well as adherence of plasma cells to other bone marrow elements, preventing aspiration. Evaluation by flow cytometry is commonly performed on second pull aspirates, which carry a greater risk of hemodilution. At the same time, flow cytometry is well known to underestimate both plasma cell content and total white blood cells, due to various technical factors and properties of the cells [32, 33].

Although the overall trend was clear as described above, there was also considerable unexplained variation between the measurements, quantified by R^2^-values of <0.5 for both comparisons (Fig 3). This is consistent with random sampling from a patchy disease process, equally affecting bone marrow core biopsies and aspirates; and varying degrees of bone marrow aspirate hemodilution between samples.

## Discussion

MRD negativity after initial therapy is an important predictor of PFS and OS in patients with multiple myeloma [5–8]. Tracking of clonal V(D)J rearrangement sequences by NGS is highly sensitive and does not require immediate analysis of fresh samples by an expert laboratory [13–15]. Variable rates of identifying clonal V(D)J sequences at baseline remain a challenge for NGS-based assays, for reasons that are poorly understood. We show that clonality detection rates of at least 95% are feasible in multiple myeloma when the sample quality is good, and describe how disease biology and sample quality together influence the probability of clonality detection.

Somatic hypermutation of the immunoglobulin variable regions has been a major obstacle to molecular MRD testing in multiple myeloma. Extensive SHM can interfere with PCR primer annealing, resulting in less effective amplification of the rearranged V(D)J sequence and making the clonal sequence indistinguishable from the background of normal polyclonal B-cells [30, 34]. SHM is a challenge particularly in plasma cell disorders due to the high degree of SHM in post germinal center B-cells. This is in contrast to for example B-lymphoblastic leukemia, where the malignant cells have not yet undergone SHM [15, 35, 36]. However, it has not been clear what role SHM plays in the context of modern NGS-based clonality assays. Importantly, we show here that modern NGS-based assays largely overcome the limitations imposed by SHM, with clonality detection rates of 95% in samples with verified presence of tumor DNA.

Low tumor cell content was the main reason for failure to identify a clonal V(D)J sequence in this study. Two lines of evidence point to hemodilution of bone marrow aspirates as an important explanation. Firstly, we describe progressively lower plasma cell content with each pull of bone marrow aspirate. This is consistent with clinical experience and previous studies showing increased hemodilution in later aspirate pulls [32, 33]. Bone marrow aspirates for research are commonly the final pull, and involve a greater volume, typically 10–20 ml. Secondly, we compared our results from research samples with data from all the patients at our institution who had baseline V(D)J sequencing done by the LymphoTrack assays as part of routine clinical workup (using early pull aspirates)[18]. The clinical samples could be separated into two groups on the basis of bone marrow plasma cell infiltration: Samples with more than 5% plasma cells had a 97% clonality detection rate, which fell to ~70% in samples with less than 5% plasma cells. Plasma cell infiltration in a bone marrow aspirate smear (first pull) was also predictive of clonality detection in our study, but considerable overlap between groups precluded the definition of meaningful cut-offs. By comparison, direct measurement of tumor DNA using myTYPE was a strong predictor. Taken together, this supports an important role for hemodilution as a reason for low tumor cell content and failure to identify a clonal sequence, particularly in research samples. Plasma cell infiltration on aspirate smears can be a good estimate of tumor cell content in early pull samples, but are less accurate for later pulls.

Patients with lambda-restricted multiple myeloma had increased probability clonality detection; mainly because clonal *IGK* rearrangements were identified more frequently in this group. We propose two explanations for this. Firstly, the *IGK* genes of lambda-restricted plasma cells contain a larger number of rearrangements than their kappa-restricted counterparts. Lambda light chain expression requires a functional rearrangement of the lambda light chain (*IGL*) gene, which happens after both *IGK* alleles have been inactivated. Inactivation results from deletion of regulatory elements, through rearrangement of the Kde region downstream of C_K_ with one of two partner regions: V_K_ in naïve *IGK* alleles, or the intronic region between J_k_ and C_k_ in the setting of a pre-existing VJ rearrangement [29, 31]. Conversely, kappa-restricted plasma cells by definition have one functional *IGK* allele. The second allele can either be in germline configuration (~50% of cases) or inactivated as described above [29, 30, 37]. The end result is 2–4 unique *IGK* rearrangements in lambda-restricted cells, while kappa-restricted cells most commonly have one or two. Because clonality detection is partly a numbers game, this provides an advantage to detect lambda-restricted clones. Finally, we show dramatically less SHM of clonal V_K_-rearrangement sequences from lambda-restricted cases as compared with kappa-restricted, consistent with previous smaller studies [29, 30]. Recruitment of activation-induced cytidine deaminase (AID) is necessary for SHM and depends on active transcription and enhancer elements, both of which are lacking in inactivated *IGK* alleles [29]. While the increased detection rate of *IGK* rearrangements without SHM provides additional potential targets for tracking, the resulting sequence will be closer to the germline and therefore more likely to occur in an independent clone by chance. Future studies are needed to better determine how the suitability of a clonal sequence for tracking is affected by SHM and other factors.

Baseline clonality detection rates vary between 80 and 97% in the published literature, using various commercially available and in-house assays [5–8, 11, 17–21]. We observed the same magnitude of difference within this study, between our whole cohort and the subset of good quality samples. Given the potential for variation due to sample quality alone, and lacking information about these aspects in many clinical trial reports, comparing assay performance between studies is unlikely to yield valid conclusions.

Strengths of our study include a large sample size from a single center, representing all disease stages (i.e. smoldering myeloma, newly diagnosed multiple myeloma, and relapsed multiple myeloma). Sequencing with LymphoTrack was done by the assay manufacturer according to a standardized protocol, and laboratory personnel were blinded to patient data. Using myTYPE as a molecular control allowed us to separate the samples into two groups with higher versus lower tumor DNA content. We emphasize the implications of myTYPE positivity, because this demonstrates the presence of tumor DNA at a level that should be detectable by LymphoTrack, unless other factors are interfering. Negative myTYPE is less informative, because each tumor contains a unique combination of clonal and subclonal somatic variants, and the detection threshold for targeted NGS assays is different depending on the variant type (e.g. SNV and CNV) [23, 38–40]. This is consistent with our the clonality detection rate of 70% in myTYPE negative samples; affirming that clonal V(D)J sequences can be identified in samples containing low levels of DNA, albeit with a lower probability of success. Weaknesses include lack of data on the number of bone marrow aspirate pulls obtained before the aliquot that was analyzed, and lack of a precise quantitative measurement of tumor cell content (myTYPE provides only a qualitative assessment). Furthermore, only samples from a single center were analyzed. Finally, most of the bone marrow samples used in this study were unsorted mononuclear cells, reflective of standard operating procedures in the research setting at the time of sample collection.

The probability of clonality detection in baseline samples is determined by two factors: The abundance of clonal cells (i.e. tumor cell content), and the degree to which clonal sequences can be amplified by the assay (which is negatively affected by SHM). Based on this, we hypothesize that increasing the tumor cell content in samples as much as practically possible (i.e., optimal bone marrow aspirates and enrichment of CD138+ plasma cells) may compensate for SHM and improve clonality detection rates beyond 95%. Another approach to improve clonality detection rates is to use a method that does not rely on specific PCR primers, to avoid the amplification bias due to SHM. For example, the mRNA-based 5’ RACE method only requires priming in the constant region and is currently used for unbiased profiling of B- and T-cell receptor repertoires [41]. The ARTISAN PCR (Anchoring Reverse Transcription of Immunoglobulin Sequences and Amplification by Nested PCR) approach was developed specifically for B-cell clonality testing based on the same principles [42]. Despite unbiased identification of clones, a challenge with these approaches is the lack of validation data for MRD tracking, and their sensitivity may be lower than that of current amplicon-based assays. In an attempt to reconcile these two approaches, we have encouraging preliminary data from combining RNA-based clonality detection followed by LymphoTrack for MRD assessment. MRD testing strategies that draw on the strengths of different technological platforms will be an attractive avenue to explore in the future.

If SHM influences PCR efficiency during baseline clonality assessment, it is important to consider that these technical conditions would be unchanged at the time of MRD testing. Currently the impact of PCR efficiency on the results and interpretation of MRD testing is not known, but in theory this is a potential bias of any amplicon-based assay (i.e. all commonly used NGS-based MRD assays). Better understanding and adjustments for such effects may increase the predictive power of MRD testing results. Reassuringly, any under-estimation of true disease burden resulting from such effects are already factored into the results of clinical trials, and therefore does not alter the fact that MRD negativity at 10^−6^ currently is (one of) the strongest prognostic factor of superior clinical outcomes in multiple myeloma[5, 6, 8, 43].

In conclusion, we have shown that identification of clonal V(D)J sequences with the LymphoTrack NGS assays largely overcome the technical challenges previously imposed by SHM in multiple myeloma, with success rates of at least 95% being feasible in samples of good quality. Bone marrow aspirate hemodilution resulting in low tumor cell content emerges as the main reason for failure to identify a clonal sequence. This is particularly true for the late pulls that are commonly obtained for research purposes. With diagnostic assays competing for high quality tumor samples, optimal bone marrow sampling and processing becomes increasingly important.

## Supporting information

S1 Appendix Supplemental methods. (DOCX)

S1 Fig LymphoTrack assay performance in dilution experiments. Dilution series of clonal control DNA in tonsil DNA for each LymphoTrack assay in 3–5 replicates. The measured percentage of clonal reads (y-axis) is plotted against the percentage of clonal DNA in the input material (x-axis). **A:** *IGH* FR1; **B:** *IGH* FR2; **C:** *IGH* FR3; **D:** *IGH* Leader; **E:** *IGK*. All assays showed linear performance with R^2^>0.98. At 2.5% dilution, the expected clonal sequence was detected well above the polyclonal background by all assays.
(PDF)

S2 Fig *IGH* framework 1–3 clonality flowchart. (PNG)

S3 Fig *IGK* clonality flowchart. (PNG)

S4 Fig *IGH* Leader clonality flowchart. (PNG)

S1 Table Target coverage requirements for different limits of detection. For each limit of detection, we report the minimum number of reads required to detect clones within ±20% of its true value with 95% confidence and 99% power.
(XLSX)

S2 Table Validation of LymphoTrack against capillary electrophoresis in clinical samples. A total of 59 samples from a variety of clinical materials (peripheral blood, bone marrow aspirate and formalin-fixed paraffin embedded tissue) were analyzed for V(D)J sequence clonality using LymphoTrack (NGS) as described in the S1 Fig legend, as well as the IdentiClone capillary electrophoresis (CE) assays (Invivoscribe) on the ThermoFischer 3500 capillary platform. Test performance is reported for the NGS assay, using the results of CE as gold standard. PPA, positive percentage agreement; NPA, negative percentage agreement. *57 samples evaluable by NGS, 46 by CE; **56 samples evaluable by NGS, 57 by CE.
(XLSX)

S3 Table Univariate regressions not included in Table 3. (XLSX)

S4 Table Expression of selected cell surface markers by flow cytometry. For each flow marker, we show the percentage of samples that are positive, as well as the number of positive and negative samples. We used chi^2 tests to check if flow marker positivity differs according to the detection of clonality by V(D)J sequencing.
(XLSX)

S5 Table Clonal kappa gene rearrangement detected in lambda vs kappa myeloma. Shows the most abundant rearrangement in each sample where clonality was detected. “Undetermined” denotes a kappa gene sequence that could not be confidently mapped to a known germline region.
(XLSX)

S1 File Raw data underlying main analysis. (CSV)

S2 File Raw data underlying in-depth analysis of *IGK* rearrangements. (CSV)
