# Supplementary material for: Baseline identification of clonal V(D)J sequences for DNA-based minimal residual disease detection in multiple myeloma
Source: PLoS One. 2019 Mar 22;14(3):e0211600. doi: 10.1371/journal.pone.0211600 (PMC6430394; doi:10.1371/journal.pone.0211600)
Supplement: S1 Appendix — (DOCX) [file pone.0211600.s001.docx]

# Baseline identification of clonal V(D)J sequences for DNA-based minimal residual disease detection in multiple myeloma

## Supplemental methods

**LymphoTrack assay validation**

Validation experiments were performed to determine the limit of detection of the assays, i.e. the lowest concentration of clonal DNA that can be distinguished from a polyclonal background. This was based on dilution series of clonal cell line DNA in polyclonal tonsil DNA. As clonal control DNA, IVS-0013 was used for the *IGH* assays, and IVS-0030 for *IGK*. Clonality was called if the lower limit of the 95 % confidence interval for the clonal control was greater than the % reads for the most abundant sequence in the polyclonal background. Algorithms to determine sample clonality at various sequencing depths were developed accordingly, and validated in a number of de-identified clinical samples. Experiments were done using the ‘Research Use Only’ assay, with sequencing and data analysis as described in the main methods section.

Minimum sequencing depth requirements to achieve a given limit of detection were estimated within ±20% of its true value with 95% confidence and 99% power using a normal-approximate z test by the SAS program (SAS/STAT^®^ 9.2 User’s guide The POWER Procedure).

**myTYPE short read alignment**
Sequence data were demultiplexed using CASAVA. Short insert paired-end reads were aligned to the GRCh37 reference human genome with 1000 genomes decoy contigs using BWA-mem[1].

**Somatic substitutions**Single nucleotide variants (SNVs) were called using three independent mutation callers: CaVEMan (<http://cancerit.github.io/CaVEMan/>) [2, 3], Strelka2 [4] and Mutect [5].

All SNVs from individual callers were then merged to a single VCF-file containing the combined evidence for each putative SNV. In addition to the variant filtering flags specific to each caller, CaVEMan variant filtering flags were applied to merged vcfs. Subsequently, variant annotation was done using Ensembl v74 using VAGrENT [6] and VEP [7] based on canonical transcripts.

To remove sequencing artifacts and likely germline variants (SNPs), we applied the following filters. Variants failing to fulfill one or more criteria were filtered out.

1. Passed by at least one out of the three variants callers
2. At least 5 reads supporting the alternative allele (read counts calculated from BAM files using cgp alleleCounter package and default settings: https://github.com/cancerit/alleleCount)
3. More than 2 % of reads covering position supports the alternative allele
4. At least one read supporting the alternative allele on each strand
5. Not detected in 16 unmatched normal samples sequenced by myTYPE.
6. Found in less than 2 % of individuals in large population databases, including 1000 genomes, ExAC and Gnomad.
7. Putative variants in genes containing only myeloma-associated germline variants were excluded.
8. Synonymous variants, and SNVs outside the targeted region or in the IGH locus were also excluded.

All putative SNVs passed by the above filters were manually reviewed. Each variant was evaluated based on the available evidence to belong to one of the following categories:

- ONCOGENIC: Reserved for hotspot mutations in oncogenes (e.g. *NRAS* Q61) and highly deleterious mutations in known tumor suppressor genes (e.g. *TP53* and *DIS3*), such as nonsense or splice acceptor variants.
- LIKELY: Some evidence for recurrent mutations at a specific position, or mutation in a gene region enriched for mutations. Missense mutations in myeloma tumor suppressor genes *DIS3* and *FAM46C* tend to fall into this category.
- UNKNOWN: Variants lacking clear evidence of being artifacts or SNPs, that also lack evidence for a role in the disease. These variants may be passenger mutations, but could also be SNPs or artifacts, and are therefore not considered in downstream analysis.
- SNP: Germline variant. Based on evidence from population databases and normal control data, as well as a variant allele fraction (VAF) around 0.5 or 1.0 (heteryzygous vs homozygous SNP).
- ARTIFACT: Resulting from sequencing errors rather than real somatic mutation. Typically low VAF, low target coverage and/or presence of one or more flags from mutation callers.

If manual classification of a variant was ambiguous, raw aligned reads were viewed in the integrated genomics viewer (IGV) for verification [8].

**Small somatic insertions and deletions**Small somatic insertions and deletions (indels) were identified using the same principles as for SNVs. Instead of CaVEMan, we used a modified version of Pindel (<https://github.com/cancerit/cgpPindel>) [9]. Mutect and Strelka2 were used as for SNVs.

To remove sequencing artifacts and likely germline variants (SNPs), we applied the following filters. Variants failing to fulfill one or more criteria were filtered out.

1. Passed by at least one out of the three variants callers
2. More than 2 % of reads covering position supports the alternative allele, based on the mean of variant allele frequency reported by the callers.
3. Not detected in 16 unmatched normal samples sequenced by myTYPE.
4. Found in less than 2 % of individuals in large population databases, including 1000 genomes, ExAC and Gnomad.
5. Putative variants in genes containing only myeloma-associated germline variants were excluded.
6. Synonymous variants, and SNVs outside the targeted region or in the IGH locus were also excluded.

All putative indels passed by the above filters were manually reviewed using the same principles as for SNVs.

**Structural rearrangements**
Given the smaller fragment insert sizes in targeted capture, the 100bp paired-end reads were trimmed to 50bp from the 3’ end of the read for better discover of in structural rearrangements. Alignment on the trimmed reads was performed as previously described and structural rearrangements were detected by an in house algorithm, BRASS [https://github.com/cancerit/BRASS], which first groups discordant read pairs that span the same breakpoint and then using Velvet de novo assembler [10] performs local assembly within the vicinity to reconstruct and determine the exact position of the breakpoint to nucleotide precision. All calls having supported by less than 5 reads were excluded. Additionally, translocations in which either of the breakpoints is involved with the IGH locus and all deletions, inversions and tandem-duplications involving the IGH locus were excluded for downstream analysis.

Additionally, an orthogonal pipeline using Delly (Version: 0.7.6) [11] was used to identify structural rearrangements. Delly was run on each tumor sample using an unmatched control sample and only those calls classified as “PASS” by Delly were retained. All calls identified in the unmatched normal were also filtered. Additionally, for translocations, only those calls having at least 6 spanning reads and 2 junction reads or at least 30 spanning reads were retained. As previously described for BRASS, translocations in which either of the breakpoints is involved with the IGH locus and all deletions, inversions and duplications involving the IGH locus were excluded for downstream analysis. All calls from Delly and BRASS were further filtered for false positives using average MAPQ, CIGAR Match length and number of reads supporting the SV. MAPQ filter is the average mapping quality of all the reads supporting the SV. We employed a MAPQ threshold of at least 30. CIGAR Match length is the average match length in the CIGAR string of all the reads supporting the SV. We used a threshold of 60 for CIGAR Match length. Lastly, supporting reads is the number of reads supporting the SV. The threshold used for support was at least 30 reads.

The resulting calls retained after the described filters were manually curated.

**Copy number aberrations**CNVKit[12] was used to identify somatic copy number aberrations in the data. To negate sample specific biases in CNV analysis, all 16 control samples were combined into a pooled reference. Each tumor sample is then compared with the pooled reference to identify somatic Copy Number Aberrations (CNA) in each sample. CNVKit corrects for biases in regional coverage and GC content, according to the given reference before calculating the log-ratios between the built pooled reference and tumor. Subsequently, Circular Binary Segmentation (CBS) algorithm is applied to obtain the log2fold change values.

Plots of genome-wide log2fold change values for each patient were used as basis for manual identification of CNVs.

**References**

1. Li H, Durbin R. Fast and accurate long-read alignment with Burrows-Wheeler transform. Bioinformatics. 2010;26(5):589-95. Epub 2010/01/19. doi: 10.1093/bioinformatics/btp698. PubMed PMID: 20080505; PubMed Central PMCID: PMCPMC2828108.

2. Nik-Zainal S, Van Loo P, Wedge DC, Alexandrov LB, Greenman CD, Lau KW, et al. The life history of 21 breast cancers. Cell. 2012;149(5):994-1007. Epub 2012/05/23. doi: 10.1016/j.cell.2012.04.023. PubMed PMID: 22608083; PubMed Central PMCID: PMCPMC3428864.

3. Jones D, Raine KM, Davies H, Tarpey PS, Butler AP, Teague JW, et al. cgpCaVEManWrapper: Simple Execution of CaVEMan in Order to Detect Somatic Single Nucleotide Variants in NGS Data. Curr Protoc Bioinformatics. 2016;56:15 0 1- 0 8. doi: 10.1002/cpbi.20. PubMed PMID: 27930805.

4. Kim S, Scheffler K, Halpern AL, Bekritsky MA, Noh E, Källberg M, et al. Strelka2: Fast and accurate variant calling for clinical sequencing applications. bioRxiv. 2017. doi: 10.1101/192872.

5. Cibulskis K, Lawrence MS, Carter SL, Sivachenko A, Jaffe D, Sougnez C, et al. Sensitive detection of somatic point mutations in impure and heterogeneous cancer samples. Nat Biotechnol. 2013;31(3):213-9. Epub 2013/02/12. doi: 10.1038/nbt.2514. PubMed PMID: 23396013; PubMed Central PMCID: PMCPMC3833702.

6. Menzies A, Teague JW, Butler AP, Davies H, Tarpey P, Nik-Zainal S, et al. VAGrENT: Variation Annotation Generator. Curr Protoc Bioinformatics. 2015;52:15 8 1-1. Epub 2015/12/19. doi: 10.1002/0471250953.bi1508s52. PubMed PMID: 26678383.

7. McLaren W, Gil L, Hunt SE, Riat HS, Ritchie GR, Thormann A, et al. The Ensembl Variant Effect Predictor. Genome Biol. 2016;17(1):122. doi: 10.1186/s13059-016-0974-4. PubMed PMID: 27268795; PubMed Central PMCID: PMCPMC4893825.

8. Thorvaldsdottir H, Robinson JT, Mesirov JP. Integrative Genomics Viewer (IGV): high-performance genomics data visualization and exploration. Brief Bioinform. 2013;14(2):178-92. doi: 10.1093/bib/bbs017. PubMed PMID: 22517427; PubMed Central PMCID: PMCPMC3603213.

9. Raine KM, Hinton J, Butler AP, Teague JW, Davies H, Tarpey P, et al. cgpPindel: Identifying Somatically Acquired Insertion and Deletion Events from Paired End Sequencing. Curr Protoc Bioinformatics. 2015;52:15 7 1-2. doi: 10.1002/0471250953.bi1507s52. PubMed PMID: 26678382.

10. Zerbino DR, Birney E. Velvet: algorithms for de novo short read assembly using de Bruijn graphs. Genome Res. 2008;18(5):821-9. Epub 2008/03/20. doi: 10.1101/gr.074492.107. PubMed PMID: 18349386; PubMed Central PMCID: PMCPMC2336801.

11. Rausch T, Zichner T, Schlattl A, Stutz AM, Benes V, Korbel JO. DELLY: structural variant discovery by integrated paired-end and split-read analysis. Bioinformatics. 2012;28(18):i333-i9. doi: 10.1093/bioinformatics/bts378. PubMed PMID: 22962449; PubMed Central PMCID: PMCPMC3436805.

12. Talevich E, Shain AH, Botton T, Bastian BC. CNVkit: Genome-Wide Copy Number Detection and Visualization from Targeted DNA Sequencing. PLoS Comput Biol. 2016;12(4):e1004873. Epub 2016/04/23. doi: 10.1371/journal.pcbi.1004873. PubMed PMID: 27100738; PubMed Central PMCID: PMCPMC4839673.
