# Supplementary figures and images for: Baseline identification of clonal V(D)J sequences for DNA-based minimal residual disease detection in multiple myeloma
Source: PLoS One. 2019 Mar 22;14(3):e0211600. doi: 10.1371/journal.pone.0211600 (PMC6430394; doi:10.1371/journal.pone.0211600)

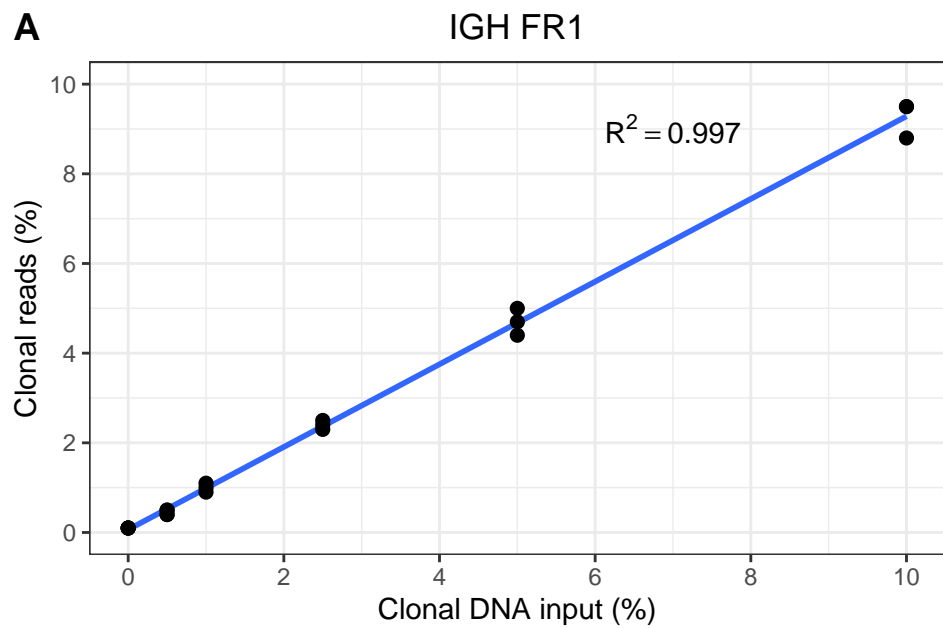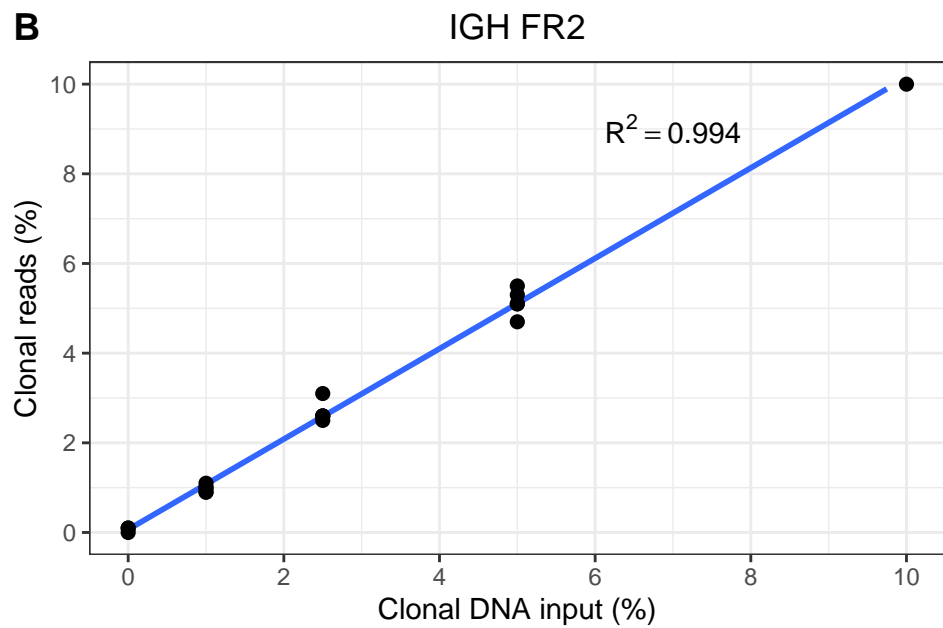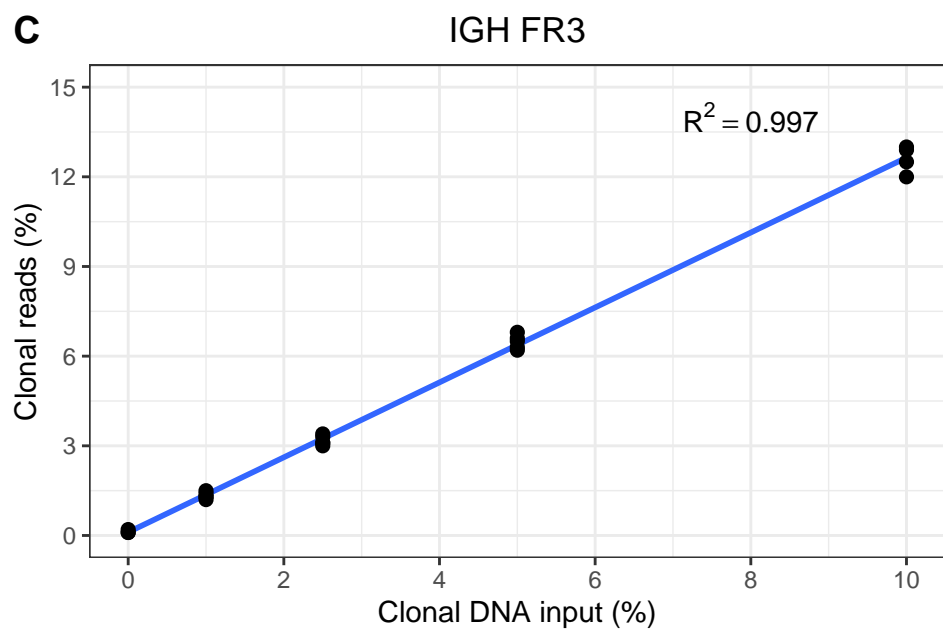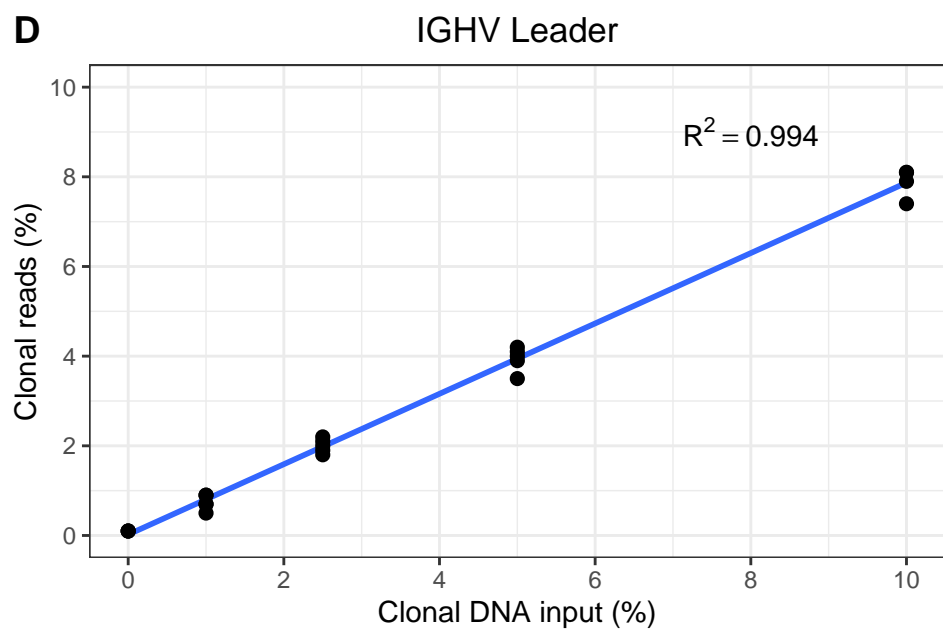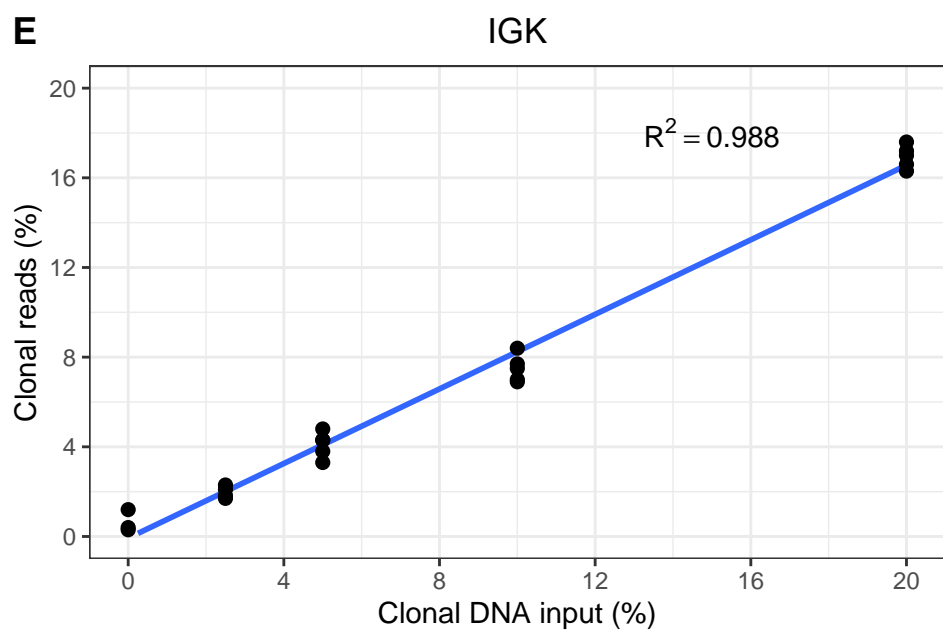

Supplement: S1 Fig — Dilution series of clonal control DNA in tonsil DNA for each LymphoTrack assay in 3–5 replicates. The measured percentage of clonal reads (y-axis) is plotted against the percentage of clonal DNA in the input material (x-axis). A: IGH FR1; B: IGH FR2; C: IGH FR3; D: IGH Leader; E: IGK. All assays showed linear performance with R2>0.98. At 2.5% dilution, the expected clonal sequence was detected well above the polyclonal background by all assays. (PDF) [file pone.0211600.s002.pdf]

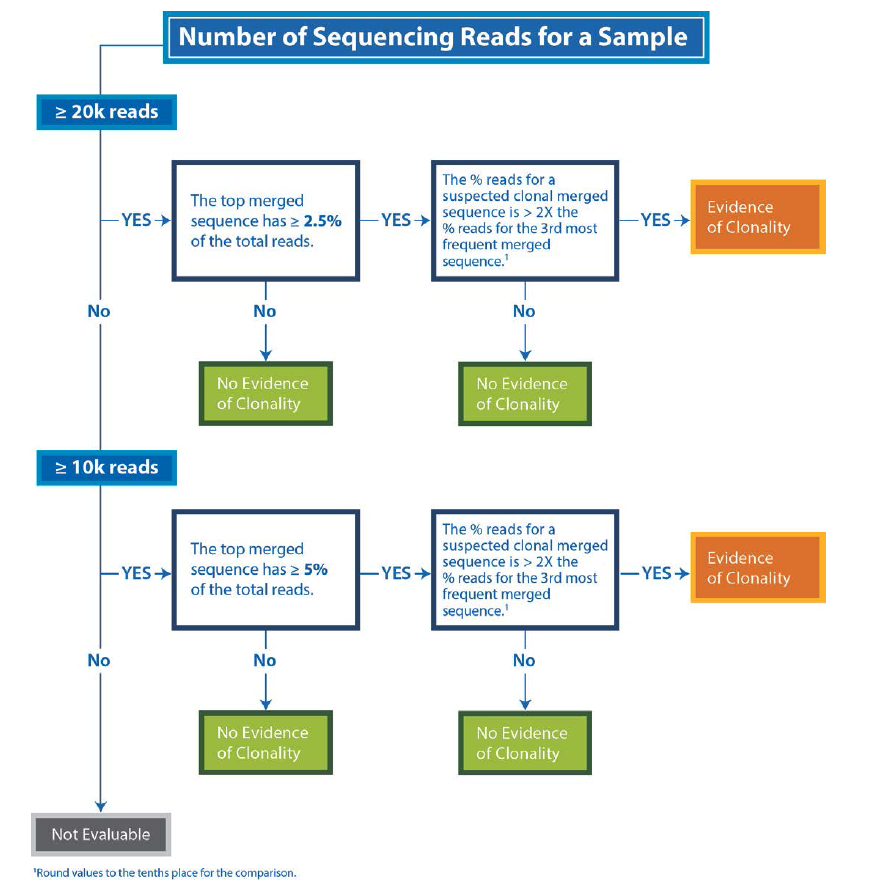

Supplement: S2 Fig — (PNG) [file pone.0211600.s003.png]

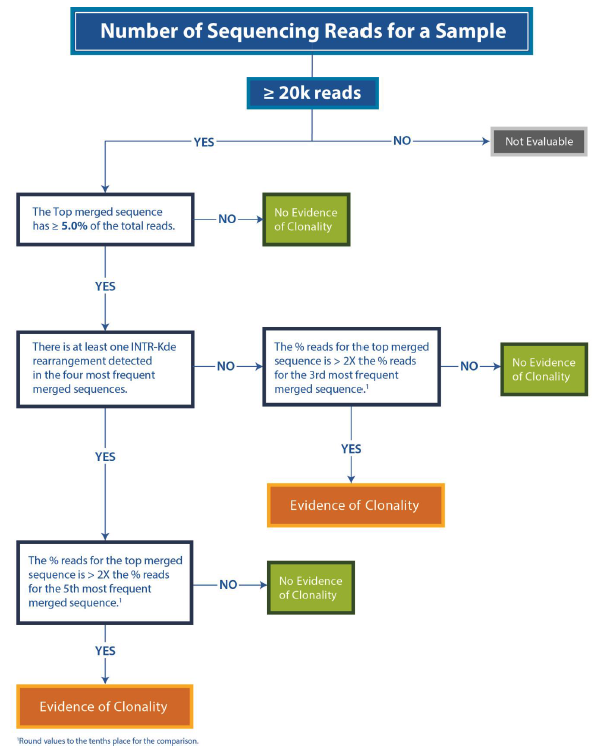

Supplement: S3 Fig — (PNG) [file pone.0211600.s004.png]

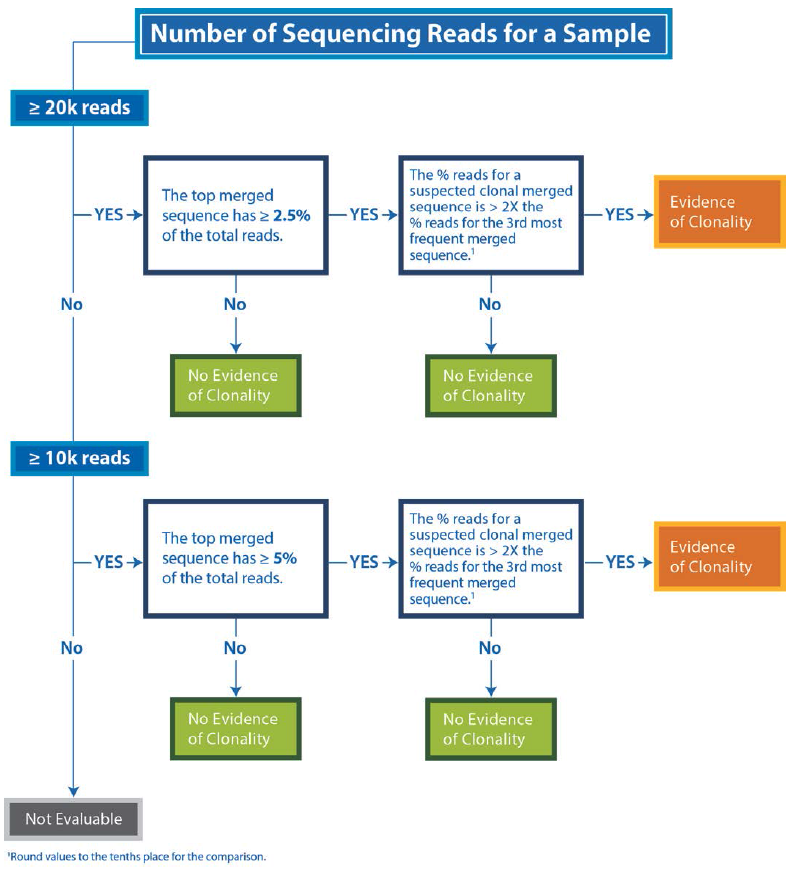

Supplement: S4 Fig — (PNG) [file pone.0211600.s005.png]
